# Personal preferences of participation in fall prevention programmes: a descriptive study

**DOI:** 10.1186/s12877-020-01586-9

**Published:** 2020-05-28

**Authors:** Lotte M. Barmentloo, Branko F. Olij, Vicki Erasmus, Dini Smilde, Yvonne Schoon, Suzanne Polinder

**Affiliations:** 1 Department of Public Health, Erasmus MC, University Medical Center Rotterdam, PO BOX 2040, 3000 CA Rotterdam, The Netherlands; 2 GENERO foundation, Rotterdam, the Netherlands; 3 Department of Geriatric Medicine, Radboud University Medical Center, Nijmegen, the Netherlands

**Keywords:** Accidental falls, Exercise, Prevention and control, Aging, Patient preference

## Abstract

**Background:**

Participation in fall prevention programmes is associated with lower risk of injurious falls among older adults. However participation rates in fall prevention interventions are low. The limited participation in fall prevention might increase with a preference based approach. Therefore, the aims of this study are to a) determine the personal preferences of older adults regarding fall prevention and b) explore the association between personal preferences and participation.

**Methods:**

We assessed the personal preferences of older adults and the association between their preferences, chosen programme and participation level. Nine different programmes, with a focus on those best matching their personal preferences, were offered to participants. Twelve weeks after the start of the programme, participation was assessed by questionnaire. Logistic regression was performed to test the association between preferences and participation and an ANOVA was performed to assess differences between the number of preferences included in the chosen programme and participation level.

**Results:**

Of the 134 participants, 49% preferred to exercise at home versus 43% elsewhere, 46% preferred to exercise alone versus 44% in a group and 41% indicated a programme must be free of charge while 51% were willing to pay. The combination of an external location, in a group and for a fee was preferred by 27%, whereas 26% preferred at home, alone and only for free. The presence of preferences or the extent to which the programme matched earlier preferences was not associated with participation.

**Conclusion:**

Despite the fact that preferences can vary greatly among older adults, local programmes should be available for at least the two largest subgroups. This includes a programme at home, offered individually and for free. In addition, local healthcare providers should cooperate to increase the accessibility of currently available group programmes.

## Background

Fall-related injuries have a substantial impact on the quality of life of individuals and on health care costs, making them a major public health problem [1, 2]. More than one-third of community-dwelling older adults, aged ≥65 years fall each year [3, 4]. In 70% of the falls, medical treatment is required. In the year 2018, within the Netherlands, falls among older adults resulted in 108.000 emergency department visits. In 70% of these visits older adults suffered from a severe injury and 33% required hospital admission. Eventually falls among older adults led to 4.396 deaths [3]. The number of fall accidents is rising, partly due to the aging population and it is expected that this rise will continue [3, 5, 6].

Many fall prevention interventions have been developed, with attention to different risk areas. Movement only interventions, generally consisting of movement or balance training, have shown to be associated with lower risk of injurious falls [7]. Multifactorial fall prevention interventions focus on more than one of the following risk areas: mobility and balance, safety in and around the house, medication use, vitamin D and vision impairment [7, 8]. Within fall accidents multiple of these areas are important, which is why multifactorial interventions are more effective in reducing falls and fall risk [7–10].

However, participation rates in fall prevention interventions, single or multifactorial, are low [11]. It is estimated by healthcare professionals that only 0–40% of older adults are reached for fall risk detection [12] and older adults that are reached are mainly those that indicate concerns themselves [13]. However, most older adults are either not aware of their fall risk [14], or they are not inclined to participate in fall prevention [15]. Personal factors, such as a low perception of the personal relevance of fall prevention programmes, and transportation problems are among the reasons to reject fall prevention interventions, even when they are offered [16–18]. Individually tailored fall prevention programmes might increase the chance that older adults will like and enjoy the programme, which has a positive influence on participation rates and active participation [18–21].

Existing studies mostly focus on barriers and motivators or the attitudes older adults have towards fall prevention. A few studies have reported programme preferences, showing that older adults seem to favour programmes with social contacts, of low intensity, free of charge and that are home based [20, 22–24]. When asked what kind of fall risk reducing programme they would be willing to participate in, Dutch older adults seem to prefer a programme consisting of exclusively home visits above programmes in their neighbourhood, by television, internet or telephone [25]. Nevertheless, these preferences or the willingness to participate do not guarantee actual participation. Besides, having a choice of interventions and programmes tailored to persons’ needs are mentioned as facilitators by older adults to participate in fall prevention programmes [22]. However, these studies did not investigate whether participation rates actually increase as a result. Therefore, in our study, we offered a wide variety of preference-based fall prevention programmes in order to investigate whether such an approach could stimulate participation. Therefore, the aims of this study are to determine; a) what the personal preferences of older adults are in participating in a fall prevention programme, and b) if there is an association between personal preferences and participation.

## Methods

This study was conducted from June 2017–December 2018 among community-dwelling adults aged ≥65 years, living in the city of Breda, in the Netherlands. Within this study, older adults received an overview of the fall prevention programmes available in their neighbourhood, highlighting those best matching their own preferences. Older adults were free to choose any programme they preferred, even if it was a poorer match to their previously indicated preferences. A questionnaire was administered at baseline and 12 weeks after commencement of the chosen programme. Not understanding the Dutch language, having dementia and living in a residential care facility were exclusion criteria. All participants provided informed consent. The medical ethics committee of Erasmus MC, University Medical Center Rotterdam reviewed the study and cleared ethical approval (number 2017–139).

### Fall prevention programmes

Fall prevention programmes were offered with an integrated neighbourhood approach to achieve a better balance between community-dwelling older adults in need for care and local healthcare givers [26, 27]. To achieve this balance, for every neighbourhood a profile was developed, relevant stakeholders were approached and different meetings were organized with these stakeholders to discuss the implementation of the programmes. Among the stakeholders were local healthcare providers, organizations representing older adults, volunteers, and representatives of local initiatives for older adults [28]. They partly facilitated the recruitment, in addition to the recruitment of the research team, and offered the fall prevention programmes. With this collaboration, older adults could participate in a programme of their own choice promoted and provided by a local healthcare provider (e.g. physiotherapist). Besides, due to this neighbourhood approach, participants could choose a programme in a close proximity of their home. This reduced travel distance, an important barrier. A total of nine exercise-based fall prevention programmes were offered in the city of Breda. The number of programmes available within each neighbourhood differed, but there was a minimum of four programmes per neighbourhood. The programmes available on offer were ‘In balans’, ‘Vallen verleden tijd’, ‘Otago’, ‘Zicht op evenwicht’, ‘Samen door’, ‘Senior Stap’, ‘Valanalyse’, senior fitness, and individual physiotherapy [9, 10, 29–33]. More information about the programmes can be found in Table 1. The programmes are a mix of evidenced based and non-evidenced based programmes in order to provide a varied offer of fall prevention programmes within each neighbourhood. The options ‘Otago’ and ‘Zicht op evenwicht’ were both offered individually and based on the evidence based options. Therefore, within our analyses these programmes are grouped together. The focus of this study is not the effect of the programmes on falls or fall risk, but exploring the preferences older adults had and how this was associated with participation.

**Table 1:** Available programmes

Programme	EB	HCW involved	Preferences	Content	Main focus
In Balans (in balance)	Yes	Physiotherapist	1 = Location, 2 = Group, 3 = Pay, 4 = Fixed, 5 = Low, 6 = Social, 7 = Together	Exercise training and information sessions	Increase risk awareness and improve balance, mobility, physical fitness and self-confidence
Vallen verleden tijd (falls in the past)	Yes	Physiotherapist	1 = Location, 2 = Group, 3 = Pay, 4 = Fixed, 5 = High, 6 = Social, 7 = Together	Obstacle course, sports and games and fall techniques	Improve mobility and reduce fear of falling
Otago	Yes	Physiotherapist	1 = Home & Location, 2 = Individually, 3 = Free, 4 = Own, 5 = Low, 6 = Sportive, 7 = Together	Leg and balance exercises and a walking program	Improve muscle strength and balance
Zicht op evenwicht (a matter of balance)	Yes	Physiotherapist	1 = Home & Location, 2 = Individually, 3 = Free, 4 = Own, 5 = Low, 6 = Sportive, 7 = Together	Information and behavior change by cognitive behavioral principles together with exercises	Reduce fear of falling
Senior step	No	Alone	1 = Home, 2 = Individually, 3 = Free, 4 = Own, 5 = Low, 6 = Sportive, 7 = Separate	Instruction book with exercises	Improve balance, mobility and strength
Samen door (go together)	No	Volunteer	1 = Home, 2 = Individually, 3 = Free, 4 = Own, 5 = Low, 6 = Sportive, 7 = Together	Easy to perform exercises addressed to the needs of the participant	To be more independent
Valanalyse (fall analyses)	No	Occupational therapist	1 = Home, 2 = Individually, 3 = Free, 4 = Own, 5 = Low, 6 = Sportive, 7 = Together	Risk assessment and a tailored advice. Besides exercise training, risk factors like medication are taken into account.	Reduce the risk of falling
Senior fitness	No	Physiotherapist	1 = Location, 2 = Group, 3 = Pay, 4 = Fixed, 5 = Low, 6 = Social, 7 = Together	Exercise training	Improve balance and mobility
Individual physiotherapy	No	Physiotherapist	1 = Home & Location, 2 = Individually, 3 = Free, 4 = Own, 5 = Low, 6 = Sportive, 7 = Together	Exercise training	Improve balance and mobility

*Note: EB* Evidenced based, *HCW* Healthcare worker. Preferences presented are 1) At home or at location, 2) In a group or individually, 3) Payment necessary or for free, 4) Fixed times or own time, 5) Low or high intensity (as indicated by the healthcare provider that offers the programme), 6) Sportive or social factors, 7) Genders separated or together

### Preferences and baseline characteristics

Participants were recruited through various methods. Among them were press releases, commercials and personal contact through local healthcare professionals such as community nurses and physiotherapists. In this way, we aimed to reach as many older adults in the city of Breda as possible. Older adults that met the inclusion criteria but were not living alone or were not vulnerable participated in a separate part of the study, described in a previous publication [34]. A more detailed description of the recruitment of participants is described in earlier publications detailing the senior step programme and investigating the implementation of the senior step programme [28, 34]. Once participants applied for the study, an informed consent form was sent by mail, accompanied by questions to assess fall risk. After written informed consent was provided, participants were telephoned by a member of the research team to assess their personal preferences and an appointment was scheduled to administer a baseline questionnaire. It was intended to administer this questionnaire during a home visit. However, due to the time investment of visiting participants at home, we could not offer all participants this home visit, and the questionnaire was administered by telephone in some instances.

### Fall risk

A history of falls and problems with movement and balance are associated with a higher chance of recurrent falls [35]. The instrument used to assess fall risk in this study is based on these two factors and was assessed by three questions; 1) did you fall in the past 12 months?; 2) do you experience problems with movement and balance?; and 3) are you afraid of falling? Older adults that answered yes on question one, or on two out of the three questions, were considered as having a high fall risk. Although this test is not yet validated, it is part of the Dutch national guidelines for assessing fall risk among community-dwelling older adults [33].

### Preferences

Preferences of participating in a fall prevention programme were collected by telephone, using seven questions with two answer options. The questions posed were drafted during a focus group together with a panel of older adults, in a participatory design approach. These older adults were part of a forum, which aims to improve the quality of life and care of older adults [36]. The group consisted of older adults, mean age 73 years, with equal proportions of men and women. Some of the panel members had a background in healthcare or experiences as a patient or caregiver and others had no specific background with healthcare. The following questions to assess older adults’ preferences were formulated: Do you prefer a programme; 1) at home or at an external location? 2) individually or in a group? 3) requiring payment or do you only want to participate if it is for free? 4) at fixed times or whenever it is convenient for you? 5) at a high or low intensity? 6) with more focus on sport or more on social factors? 7) with men or women separately, or together?

### Baseline characteristics

A baseline questionnaire was assessed by a member of the research team during a home visit or by telephone. This questionnaire was a combination of the TOPICS-MDS and the EQ-5D + cognition questionnaire [37, 38]. The TOPICS-MDS is a validated questionnaire and advised for use in a geriatric population [38]. It includes items on sociodemographic characteristics, such as age, gender, living situation, marital status, country of birth, education level and diseases experienced during the last 12 months. Education was arranged in low (less than primary school, primary school, and a little more than primary school), middle (i.e. technical school, vocational education, general secondary/pre-university education), and high (i.e. college/university). For diseases a list of seventeen diseases was listed as used in the TOPIC-MDS. In addition, an option ‘other disease’ was added. Participants could indicate whether they had experienced the disease in the last 12 months. Health-related quality of life was assessed by the three level EuroQol instrument (EQ-5D + cognition), in which the domains mobility, self-care, usual activities, pain and discomfort, anxiety and depression, and cognition were included [37]. A summary score ranged from 0 (death) until 1 (full health).

### Referral

Participants were provided with flyers of all fall prevention programmes that were available in his or her neighbourhood. Participants received the flyers during the home visit or by post after the telephone call. Information on the flyers consisted of the main aim, content, duration, frequency, number of participants, location and costs of the programme. In addition, information about possible reimbursement of programme costs by health insurances was added. A member of the research team discussed the personal programme preferences, the programmes on offer in participants’ neighbourhood and the best matches between the two. After that the participant was given time to decide which programme suited them best. Two weeks later a member of the research team telephoned the participant to enquire whether the participant had chosen a programme. The participant was free to choose any of the programmes available in the neighbourhood. Once a participant had chosen a programme, a member of the research team initiated the first contact with the local healthcare provider that offered the programme. The healthcare provider then contacted the participant and made an appointment to start the fall prevention programme. In some cases participants could start straight away (e.g. individual programmes); in other cases participants received a date when their chosen programme would start in the future (e.g. for group programmes).

### Follow-up characteristics

Twelve weeks after the start of the chosen programme, a member of the research team telephoned the participant again and a follow-up questionnaire about participation was administered. Frequent participation was classified as performing exercises of the fall prevention programme daily or a few days a week during the 12-week study period. Infrequent or nonparticipation was classified as performing exercises one day a week, less than one day a week, or not at all. These classifications are described in an earlier publication of the study [34]. Furthermore experiences with and perceptions of the programme were assessed by multiple choice questions, in accordance with the guideline for medical scientific research in older adults [39]. The questions were based on expert opinions in the research team and can be found in Additional file 1.

### Statistical analyses

For baseline and follow-up characteristics, continuous variables were expressed as mean and range, dichotomous variables and preferences were expressed as number and percentage. Differences at baseline between participants with a low and high fall risk were compared using an independent t-test for continuous variables and a chi-squared test for dichotomous variables. In order to determine the correlation between participants in terms of their personal preferences, a two-tailed Pearson correlation was used. To investigate whether there was an association between baseline personal preferences and the presence of the preferences in the programme individuals participated in, Spearman Partial Correlation was performed, adjusted for fall risk. To investigate the association between particular preferences in the chosen program and participation level, logistic regression was applied where the presence of the preferences was used as independent variable and the participation frequency as dependent variable. In order to plot the number of preferences that were eventually present in the fall prevention programme against the follow-up characteristics, an ANOVA test was performed. A distinction was made between participants for which five out of seven or less preferences were present in their chosen programme, six out of seven preferences were present, or all seven preferences were present in the programme. In all analyses a *p*-value of <.05 was considered statistically significant. Analyses were performed using SPSS Statistical Data software (IBM), version 24.

## Results

**Fig. 1:**
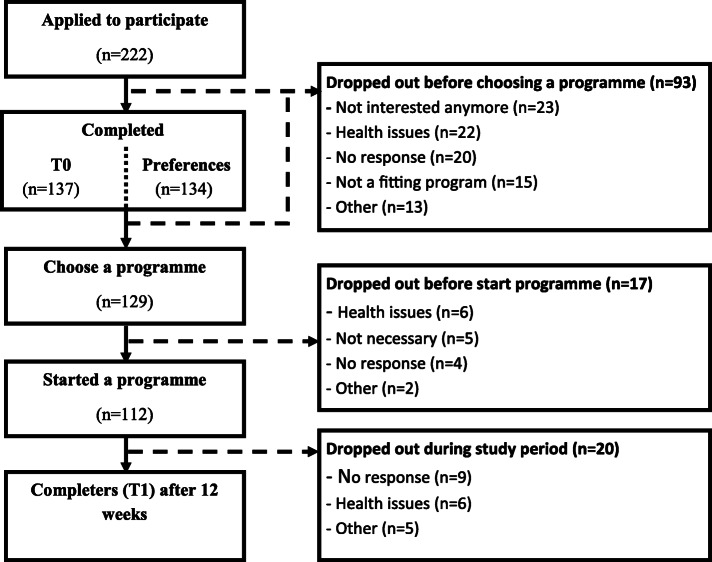
Flowchart

Besides indirect methods (commercials on local television and radio channels), potentially 3100 older adults were reached by recruitment through community nurses, flyers and other direct methods [28]. A total of 222 older adults that met the inclusion criteria were interested in following a fall prevention programme and included in the current study. Due to a dropout of 92 older adults (41%), eventually 129 (59%) indicated that they wanted to start with a particular programme. Older adults that indicated that they wanted to start with a particular programme were younger and they lived independently more often compared to the non-responders, but no difference in fall risk or gender was observed (Additional file 2). In the end, 51% of the older adults started with a programme and 42% of all older adults completed the programme (Fig. 1). From the start onwards, a total loss of 130 participants was seen. Of them, 25% were lost because the research team could not reach them (telephone not answered, etc.). Of the remaining 75%, reasons for dropout during the process were: older adults had experienced health problems which impeded participation (26%), older adults thought they did not need a fall prevention programme any more (22%), the programmes available during that time did not meet their preferences (12%) or participants had other reasons to dropout (15%).

### Baseline characteristics

Baseline characteristics were collected from 137 participants (Table 2). The mean age of participants was 80.5 years, most were women, Dutch, lived independently and were widow/widower. Most participants indicated having problems with mobility (73%) and daily activities (64%). Furthermore, 70% of the participants indicated experiencing pain and discomfort. A high fall risk was detected in 64% of the participants. Several differences between participants with a high and low fall risk were observed. Participants with a high fall risk had a lower EQ-5D utility score than those with a low fall risk (0.55 vs 0.71, *p*-value = <.001). In addition, participants with a high fall risk had more problems with mobility (85% vs 52%, p-value = <.001), self-care (52% vs 22%, p-value = .001), and daily activities (78% vs 40%, p-value = <.001) than participants with a low fall risk.

**Table 2:** Baseline characteristics (n = 137) and differences between high and low risk participants

Characteristics	Total *n* = 137	High risk (64%) *n* = 87	No/low risk (36%) *n* = 50	*P*-value
	Mean (range)	Mean (range)	Mean (range)	T-test
Age	80.6 (65–99)	80.8 (65–99)	80.3 (65–95)	.737
EQ 5D weight score	0.61 (0.13–1)	0.55 (0.13–1)	0.71 (0.18–1)	<.001
	**n (%)**	**n (%)**	**n (%)**	**Chi-Square**
Gender				.893
Men	32 (23.4)	20 (23.0)	12 (24.0)	
Women	105 (76.6)	67 (77.0)	38 (76.0)	
Country of birth				.019
Netherlands	128 (93.4)	78 (89.7)	50 (100.0)	
Other	9 (6.6)	9 (10.3)	0 (0.0)	
Education				
Low	47 (34.3)	34 (39.1)	13 (26.0)	.121
Middle	67 (48.9)	36 (41.4)	31 (62.0)	.020
High	23 (16.8)	17 (19.5)	6 (12.0)	.256
Living situation				.808
Independent	103 (75.2)	66 (75.9)	37 (74.0)	
Independent with others	34 (24.8)	21 (24.1)	13 (26.0)	
Marital status				
Married	34 (24.8)	21 (24.1)	13 (26.0)	.808
Divorced	9 (6.6)	8 (9.2)	1 (2.0)	.102
Widow/widower	81 (59.1)	52 (59.8)	29 (58.0)	.839
Unmarried	11 (8.0)	5 (5.7)	6 (12.0)	.195
Sustainably living together	2 (1.5)	1 (1.1)	1 (2.0)	.689
Diseases present				
< = 1 diseases	21 (15.3)	8 (9.2)	12 (26.0)	.009
2 diseases	34 (24.8)	18 (20.7)	16 (32.0)	.140
3 diseases	22 (16.1)	16 (18.4)	6 (12.0)	.327
4 diseases	23 (16.8)	16 (18.4)	7 (14.0)	.508
5 or more diseases	37 (27.0)	29 (33.3)	8 (16.0)	.028
Problems with				
Mobility	100 (73.0)	74 (85.1)	26 (52.0)	<.001
Self-care	56 (40.9)	45 (51.7)	11 (22.0)	.001
Daily activities	88 (64.2)	68 (78.2)	20 (40.0)	<.001
Pain/discomfort	97 (70.8)	62 (71.3)	35 (70.0)	.875
Mood	46 (33.6)	32 (36.8)	14 (28.0)	.295
Cognition	45 (32.8)	32 (36.8)	13 (26.0)	.196

*Note:* An independent t-test was used for continuous variables and a chi-squared for dichotomous variables. A *p*-value of <.05 was considered statistically significant

### Preferences

**Fig. 2:**
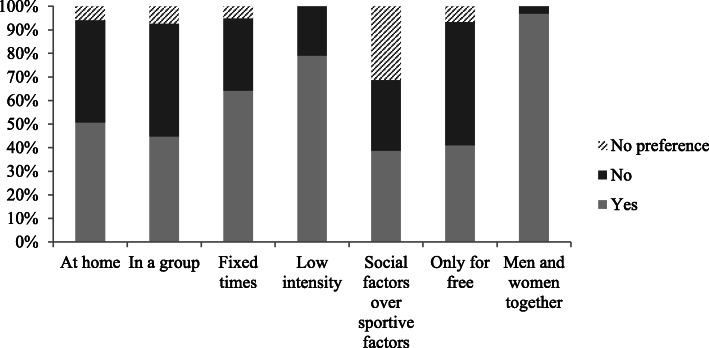
Exercise preferences of participants regarding fall prevention (*n* = 134 (men and women together *n* = 132)). Data of 3 participants is missing, since they already signed up for a fall prevention programme before entering the study

Of the 134 participants that completed the preferences questionnaire, 49% specified they preferred a fall prevention programme at home versus 43% that preferred a programme at a location outside their home. An individual fall prevention programme was preferred by 46% versus 44% that preferred the group option. Most participants were willing to pay (51%) for a fall prevention programme, although 41% of the participants indicated that they only wanted to participate in a fall prevention programme if it was free of charge. Some participants did not have a clear preference for one of the two options. An overview of other preferences can be found in Fig. 2. There was a positive correlation between participants that preferred to exercise at home and individually (*r* = .769, *p*-value = <.001). Furthermore, there was a positive correlation between participants that preferred to exercise at an external location and in a group (*r* = .764, p-value = <.001). When looking at combinations of the preferences 1) at home or at an external location, 2) alone or in a group and 3) only for free or willing to pay, two subgroups can be distinguished. Specifically, 27% of the participants preferred a programme at an external location, in a group, and for a fee whereas another 26% of the participants preferred a programme at home, alone, and free of charge. When comparing participants with a high and a low fall risk, a larger percentage of those with a high fall risk preferred to exercise at home (59% vs 34%, *p*-value = .006) and alone (56% vs 30%, p-value = .003) than those with a low fall risk. No differences in programme preferences was seen between older adults that started a fall prevention programme compared to those that did not.

### Fall prevention programmes

For 31 participants (32%), six out of seven preferences were present, and for 38 participants (40%), all preferences were present in the fall prevention programme they started. Where five or fewer preferences were met, at least four preferences were met for 19 participants (20%) and only for eight participants (8%) three or fewer preferences were met. Eventually the majority of all participants started with an individual-based fall prevention programme (63%), free of charge (63%), at home (53%). When comparing participants with a high and low fall risk, a larger percentage of those with a high fall risk started with an individual-based programme that was free of charge (71% vs 49%, *p*-value = .014) and at home (61% vs 40%, *p*-value = .025) than those with a low fall risk. An overview of the fall prevention programmes chosen by participants can be found in Table 3.

**Table 3:** Chosen fall prevention programmes (*n* = 129) and differences between high and low risk participants, exploratory data

Programme	Total (*n* = 129)	High risk (64%) (*n* = 82)	No/low risk (36%) (*n* = 47)	Chi-square
	n (%)	n (%)	n (%)	P-value
In Balance	17 (13.2)	10 (12.2)	7 (14.9)	.788
Falls in the past	16 (12.4)	7 (8.5)	9 (19.1)	.098
Otago / A Matter of balance	13 (10.1)	9 (11)	4 (8.5)	.768
Senior Step	21 (16.3)	13 (15.9)	8 (17)	1.000
Fall analysis	18 (14)	12 (14.6)	6 (12.8)	1.000
Individual physiotherapy	20 (15.5)	17 (20.7)	3 (6.4)	.042
Senior fitness	15 (11.6)	7 (8.5)	8 (17)	.163
Go together	9 (7)	7 (8.5)	2 (4.3)	.485

*Note:* A *p*-value of <.05 is considered statistically significant

When comparing the association of personal preference and characteristic of chosen programmes, some preferences showed a moderate to strong association, such as the preferences “at an external location” (*r* = .574, *p*-value = <.001), “at home” (*r* = .529, p-value = <.001) and “in a group” (*r* = .546, p-value = <.001). For the preferences high or low intensity, social or sportive factors and genders mixed or genders separated, no association was found. Other associations can be found in Table 4.

**Table 4:** Correlations between the baseline personal preferences and the presence of these preference

Preference	Spearman Partial Correlation	
	Value	Significance
**At home**	.529	<.001
**External location**	.574	<.001
**Individually**	.453	<.001
**Group**	.546	<.001
**Own time**	.339	<.001
**Fixed time**	.306	<.001
**Low intensity**	.019	.827
**High intensity**	.042	.636
**Social factors**	−.143	.108
**Sportive factors**	.050	.574
**For free**	.360	<.001
**Willing to pay**	.294	.001
**Gender separate**	.045	.616
**Gender mixed**	.047	.600

### Follow-up characteristics

**Fig. 3:**
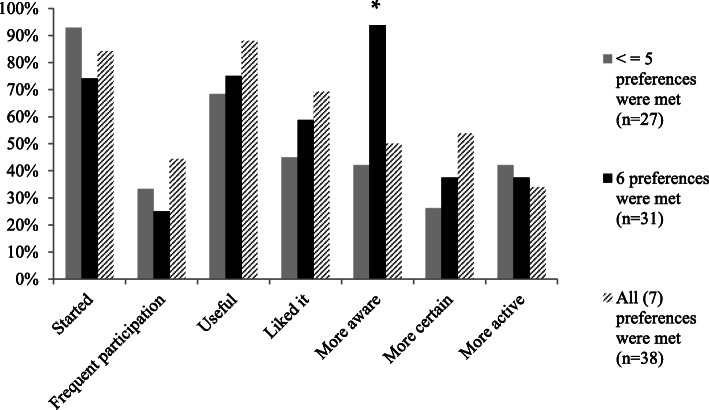
The amount of preferences present, plotted against the follow-up-characteristics. *Significant (*p*-value = <.05) difference between the groups

Frequent participation during the study period was indicated by 38% of the participants. Seventy-four percent of the participants indicated that their programme was useful, 52% that they liked the programme, 55% reported to be more aware of their fall risk, 38% reported an increased confidence in their balance, and 35% of the participants noticed a change in their level of physical activity. In Fig. 3, the number of preferences that were present in the fall prevention programme is plotted against the follow-up characteristics. A distinction was made between participants that followed a programme in which five or fewer preferences were present, six preferences were present, or all preferences were present in the chosen programme. Participants that participated in a programme in which six of their preferences were present, were more likely to be aware of their fall risk than participants for which less than six or all preferences were present in the programme (F(2, 58) = 6.452, *p*-value = .003). No statistically significant associations were observed between the presence of personal preferences in the programme, and the level of participation in the programme. In addition, no associations were found between the presence of particular preferences and participation level.

## Discussion

We found that 49% of participants prefer to exercise at home versus 43% elsewhere, 46% prefer to exercise individually versus 44% in a group, and 41% only want to participate in a programme free of charge versus 51% that is willing to pay. Two subgroups can be distinguished with these preferences, namely a subgroup that prefers a programme outside their own home, in a group and is willing to pay (27%) and a group that prefers a programme at home, alone and is not willing to pay (26%). For 38 (40%) participants, all personal preferences were present in the programme they started. However, no association was found between the number of personal preferences or a particular preference in the programme and participation level.

The personal preferences of the participants in our study varied greatly between individuals. This preference variation between older adults has also been confirmed by a previous study [20]. However, in our study, the majority of participants chose three clear preferences; mixed gender, low intensity, and at fixed times. Even though, in general, the majority of exercise programmes are offered at an external location, the majority of older adults appear to prefer a home-based programme [23–25]. In our study we found that this ‘at home’ option was more often preferred and chosen among high risk older adults. This difference between older adults at a low and high risk is supported by a study of Dorresteijn et al. in which older adults with a history of multiple falls were more likely to prefer a home-based programme [25]. In our study, 64% of the participants had a high fall risk, which is linked to a history of falls. This highlights a gap between the current offer and the preferences of the most vulnerable community-dwelling older adults. Namely, those of higher age, a lower health related quality of life and a high fall risk. Currently, the evidence based options for this population are limited and not widely offered. This problem will probably increase in the coming years due to a larger group of older adults that will continue living at home longer, making it important to meet the preferences of these more vulnerable older adults. This home-based preference of vulnerable older adults is also in line with the preference that older adults in general have about physical activity, namely that they prefer individual activity [40]. A great opportunity for home-based individual fall prevention interventions in the future could be digital interventions, such as tablet, computer or smartphone based applications. Currently studies are still investigating the effectiveness of these programmes on falls. However, considering that these programmes are based on evidence based exercises it has great potential for future fall prevention [41–43]. Furthermore, we found that more than half of the older adults are willing to pay for an intervention, which is supported by Child et al. who found that older adults are willing to pay as long as it is reasonable [19]. However, 41% still indicate that they will or cannot pay for a fall prevention programme. It is important to consider this population, since costs can be a barrier for participation in fall prevention [23]. Especially for older adults with lower socio-economic status. In order to not increase potential health differences between higher and lower socio-economic status, investments have to be made to create more opportunities free of charge.

In addition to the preferences mentioned, we can distinguish two subgroups when it comes to preferences. These groups account for half of the total cohort which makes it important to create an appropriate offer for these groups. Based on these preferences, there should be a fall prevention programme which can be performed at home, alone and for free and a programme outside the home, in a group and with a possibility for a fee. We already discussed the gap between the more vulnerable older adults and a programme at home, individually and for free. And although evidence based options for a programme in a group, at an external location and for a fee are available in the Netherlands, also here a gap arises between the programmes on offer and the preferences of the community-dwelling older adults. This problem arises because these programmes are not widely offered throughout different neighbourhoods, which causes poor accessibility. The availability of programmes within older adults’ own neighbourhood is important, since transportation problems are an important reason for older adults to reject interventions [16, 19]. In order to make these programmes more accessible they should be offered within different neighbourhoods or transportation options should be offered. This requires good coordination between the different health care providers, who sometimes see each other more as competitors, which counteracts their cooperation.

The fact that no associations were found between the presence of personal preferences in the programme and the level of participation could suggest that other factors might be more important. In addition, the preferences participants had did not seem to influence whether they eventually started a programme. This strengthens the impression that other factors have more influence on participation. Factors such as the social component of a group, a good relationship with the provider or current health status might be more important [44–46]. A review of Bunn et al. showed that factors in fall prevention programmes such as social support and interaction and the idea that a programme is beneficial facilitate participation [22]. Besides, intrinsic motivation could play a more important role in the uptake of fall prevention activities. This reasoning can be supported by the reasons why older adults participate in the first place, namely staying in good health and the fear of becoming vulnerable [18, 20]. Furthermore, this intrinsic motivation could also arise from a recent fall or multiple falls in the past, which are associated with a higher uptake of fall prevention programmes [24]. In addition, only 55% of the participants indicate that they liked the programme. Nevertheless, despite not particularly liking it, this group followed the programme for at least 12 weeks, which suggests that for example intrinsic motivation or the belief in positive health outcomes might be more important. Qualitative studies should investigate older adults’ reasons for participating in fall prevention. Despite the fact that we found some factors that were associated with the characteristics of chosen programmes, we cannot conclude that these are the most important factors for older adults to participate. In the current study, this association is strongly associated with the available offer of fall prevention programmes during the study period. To find out precisely which factors are most important for older adults, more research is needed, for example through a discrete choice experiment (DCE).

This study provides an overview of the preferences community-dwelling older adults have when it comes to participating in fall prevention programmes. A strength of the study was the participatory design approach, in which community dwelling older adults were involved as part of the research team in each phase of the study. In addition, in the current study more than six out of those seven preferences were present in the fall prevention programme started by 72% of the participants. This was achieved by stimulating healthcare providers to offer as many different programmes as possible in the various neighbourhoods. Nine different programmes were on offer, which increased the chances of a possible ‘match’. Because many preferences returned in the fall prevention program, categories of preferences met needed to be changed into seven, six out of seven and five or less preferences met. This was instead of the preferred option, namely, a weak match (1–3 preferences), moderate match (4–5 preference) and a good match (6–7 preferences). Despite this broad offer, some participants were still not able to start a programme matching their preferences during the study period. Which indicates that there is still a gap between older adults preferences and the available fall prevention programmes. This gap caused some drop-out, which is a limitation of the current study.

Of the 222 older adults that applied to participate, 112 individuals finally participated in the study. Most of these were recruited by a community nurse and only a few by self-identification, reached by flyer or commercials, and therefore we cannot look into the differences in preferences or chosen programmes between different recruitments styles. We did look at different programmes chosen by the participants, but due to the high drop-out, some of the numbers in Table 3 are small and therefore these numbers are only exploratory. A reason for drop-out that was often mentioned by older adults was ‘poor health’, which has been observed in other studies as well [18, 20], but also the delay between choosing a programme and the moment the programme started could have resulted in drop-outs among older adults. This sometimes took more than a month. In this period declining health or motivation could have taken place among participants. In addition, sometimes participants’ preferences did not match the available offer. This was mainly seen when participants preferred a group based programme, but did not want or were not able to pay for it or the programme was not offered within their neighbourhood. This high drop-out could have influenced the results. Furthermore, we classified frequent participation as daily and a few days a week. However, potential reverse effects of daily participation are not taken into account with this classification, based on the little evidence that is available on this topic [47]. Also how much participants liked the programme and thought it was useful was classified categories while qualitative data on participants experiences and perceptions could have added valuable information to the paper. Moreover, it should be taken into account that data was collected in person as well as by telephone. This difference could have impacted response choices, considering that data collected face-to-face can possibly lead to more socially desirable answers. Lastly, we are only able to make conclusions about this cohort, given that this is a rather old, Caucasian and vulnerable population with a high number of women, which raises the question of generalizability. However, this is a reflection of the older Caucasian race, especially for an older community-dwelling population.

When implementing fall prevention programmes locally, it should be taken into account that preferences can vary greatly between older adults. Local policy makers together with health care providers should arrange applicable programmes for the two largest subgroups, 1) at home, individually and free of charge and 2) outside their own home, in a group with the possibility of a fee. Another aspect that should be taken into account is the preference for a programme free of charge. Despite the fact that most older adults are willing to pay (51%) for a fall prevention programme, still 41% of the older adults are not willing or able to pay. Fall prevention programmes free of charge or completely covered by health insurance are limited, while investing in exercise based fall prevention programmes is cost-effective [48]. To prevent falls among this population it is important to offer a programme free of charge or covered by health insurances. Finally, when targeting adults with a high fall risk, the offer of programmes should mainly focus on individual programmes at home, since this is most preferred by this high risk population. Nevertheless, health care providers and local policy makers have to be careful in adopting to personal preferences of older adults because it is unknown whether programmes remain effective in reducing falls by adopting to these personal preferences. A review of Sherrington et al. showed that exercise interventions have to consist of specific exercises and a certain intensity-level to be effective [49]. Adopting to personal preferences could be at the expense of these exercises or intensity and thus reduce the effectiveness.

## Conclusion

There is a wide range of preferences when it comes to participating in a fall prevention programme. However, there is a large group that prefers a fall prevention programme at home, alone and for free and a large group that prefers a programme outside their own home, in a group and is willing to pay for it. Furthermore, older adults already at high risk for falls prefer an individual programme at home more often. In particular, the preferences location (at home or an external location) and in a group are often found in the programme participants started. However, once older adults start with fall prevention, these preferences seem less important, since there is no association between preferences returning in a programme and participation level.

## Supplementary information

**Additional file 1.** Classification of follow-up characteristics

**Additional file 2.** Baseline Characteristics of Responders and Non-Responders.

## Data Availability

The datasets used and/ or analysed during the current study are available from the corresponding author on reasonable request.

## Abbreviations

ANOVA: Analysis of variance
DCE: Discrete choice experiment
EB: Evidenced based
EQ-5D: EuroQol five dimensions
ErasmusMC: Erasmus medical center
HCW: Healthcare worker
IBM: International business machines corporation
SD: Standard deviation
SPSS: Statistical package for the social sciences
TOPIC-MDS: The older persons and informal caregivers survey minimal dataset
T0: Time point 0
T1: Time point 1
