# Supplementary material for: Personal preferences of participation in fall prevention programmes: a descriptive study
Source: BMC Geriatr. 2020 May 28;20:185. doi: 10.1186/s12877-020-01586-9 (PMC7254764; doi:10.1186/s12877-020-01586-9)
Supplement: Supplementary file 1 — Additional file 1. Classification of follow-up characteristics [file 12877_2020_1586_MOESM1_ESM.pdf]

Additional file 1 Classification of Follow-up Characteristics.

Questions	Answers	Classification
Did you got started with the fall prevention programme?	1. Yes<br>2. No	
How often did you perform actions of the fall prevention programme?	1. Daily<br>2. Multiple times a week<br>3. Once a week<br>4. Less than once a week	Daily or multiple times a week is classified as "Frequent participation".<br>Once a week or less than once a week is classified as "Non-frequent participation".
How much did you like the programme?	1. Not nice at all<br>2. A little nice<br>3. Pretty nice<br>4. Very nice	Not nice at all and a little nice is classified as "Did not like it"<br>Pretty nice and very nice is classified as "Liked it".
How useful did you find the programme?	1. Not useful at all<br>2. A little useful<br>3. Pretty useful<br>4. Very useful	Not useful at all and a little useful is classified as "Not useful".<br>Pretty useful and very useful is classified as "Useful".
Since I have participated in the falls prevention programme, I am more aware of my fall risk.	1. Strongly disagree<br>2. Disagree<br>3. Neither agree or disagree<br>4. Agree<br>5. Strongly agree	Strongly disagree, disagree and neither agree or disagree is classified as "Disagree".<br>Agree and strongly agree is classified as "Agree".
Since I have participated in the falls prevention programme, I feel myself more secure while standing and walking.	1. Strongly disagree<br>2. Disagree<br>3. Neither agree or disagree<br>4. Agree<br>5. Strongly agree	Strongly disagree, disagree and neither agree or disagree is classified as "Disagree".<br>Agree and strongly agree is classified as "Agree".
Since I have participated in the falls prevention programme, I exercise more often.	1. Strongly disagree<br>2. Disagree<br>3. Neither agree or disagree<br>4. Agree<br>5. Strongly agree	Strongly disagree, disagree and neither agree or disagree is classified as "Disagree".<br>Agree and strongly agree is classified as "Agree".
