# Supplementary material for: Personal preferences of participation in fall prevention programmes: a descriptive study
Source: BMC Geriatr. 2020 May 28;20:185. doi: 10.1186/s12877-020-01586-9 (PMC7254764; doi:10.1186/s12877-020-01586-9)
Supplement: Supplementary file 2 — Additional file 2. Baseline Characteristics of Responders and Non-Responders. [file 12877_2020_1586_MOESM2_ESM.pdf]

Additional file 2 Baseline Characteristics of Responders and Non-Responders

Characteristics	Total (n=222)	Responders (n=130)	Non-responders (n=92)	P-value
	mean ± SD	mean ± SD	mean ± SD	T-test
**Age**	79.9 ± 7.2[a]	80.7 ± 7.2	78.7 ± 7.1[b]	.039
	n (%)	n (%)	n (%)	Chi-square
**Female**	165 (74.3)	100 (76.9)	65 (70.7)	.292
**Living alone**	156 (70.3)	98 (75.4)	58 (63)	.048
**Elevated fall risk[1]**	163 (73.4)	98 (75.4)	65 (70.7)	.432

Note: SD: Standard deviation; [1] The fall risk test is determined an elevated fall risk when older adults were fallen in the last 12 months, or had mobility problems and a fear of falling. [a]: n=219, [b]: n=89

1. VeiligheidNL. Valanalyse screeningstool valrisico voor de eerstelijnszorg [fall analysis, fall risk screening tool for primary care. In dutch] https://intranet.onzehuisartsen.nl/file/download/default/A0990575496919D58AF03796C9263DFC/VNL-valanalyse-2017-ONLINE.pdf
